# Real-world feasibility of privacy-preserving, non-wearable AI for real-time fall detection with disease-specific video classification in parkinsonian syndromes: a proof-of-concept clinical study

**DOI:** 10.3389/fneur.2026.1856113

**Published:** 2026-09-18

**Authors:** Hikaru Kamo, Genko Oyama, Koichi Nagaki, Yoshio Tsuboi, Taku Hatano, Nobutaka Hattori

**Affiliations:** 1 Department of Neurology, Juntendo University Graduate School of Medicine, Bunkyo-ku, Tokyo, Japan; 2 Department of Home Medical Care System, Based on Information and Communication Technology, Juntendo University Graduate School of Medicine, Bunkyo-ku, Tokyo, Japan; 3 Department of Neurology, Fixel Institute for Neurological Diseases, University of Florida, Gainesville, FL, United States; 4 Department of Service Development, Sunwels Company Limited, Minato-ku, Tokyo, Japan; 5 Department of Health Economics, Center for Gerontology and Social Science, Research Institute, National Center for Geriatrics and Gerontology, Obu City, Aichi, Japan; 6 Neurodegenerative Disorders Collaborative Laboratory, RIKEN Center for Brain Science, Wako-shi, Saitama, Japan

**Keywords:** artificial intelligence, fall detection, Parkinson’s disease, progressive supranuclear palsy, real-world data, video analysis

## Abstract

**Background:**

Falls in Parkinson’s disease (PD) and progressive supranuclear palsy (PSP) are frequent yet often missed in clinical care. We evaluated a privacy-preserving, non-wearable AI system for real-time fall detection and exploratory video analysis in Parkinsonian syndromes.

**Methods:**

Residents of a PD-specialized facility were continuously monitored for 22–63 days using a ceiling-mounted depth camera with in-sensor edge AI. All video-confirmed falls were reviewed by two blinded raters and classified into 10 predefined operational categories. Detection performance was summarized descriptively, and fall characteristics were explored using chi-square tests with false-discovery-rate control.

**Results:**

Across 6,272 h of monitoring, 74 real-world falls were confirmed. AI sensitivity was 52.7% across the full study and 67.6% during the post-update evaluation phase, whereas conventional facility reporting identified 28.4% and 29.4% of video-confirmed falls, respectively. The corresponding positive predictive values of the AI system were 45.3% and 41.8%. Comprehensive video review established the reference count of confirmed fall events within the analyzable recordings. Exploratory video analysis suggested that falls in PD were more often classified as involving observable contexts compatible with attentional, executive, or visuospatial contributions, whereas falls in PSP were more often classified as involving inadequate weight shift or transfers. Most falls in PD and PSP occurred during routine movements without obvious external perturbations.

**Conclusion:**

Continuous, privacy-preserving depth-camera monitoring identified falls that were not captured by routine facility reporting. The observed disease-related fall patterns are exploratory and require validation in larger, independent cohorts.

## Introduction

Falls are a major concern in neurodegenerative diseases, such as Parkinson’s disease (PD) and atypical Parkinsonian syndromes, particularly in the advanced stages (1, 2). They significantly affect patients’ quality of life (QoL) and activities of daily living (ADL) (3), leading to fractures, injuries, hospitalization, fear of falling, gait disturbances, increased caregiver burden, and ultimately, mortality (4). Falls are a prominent clinical feature in PD and are considered one of the hallmark manifestations of progressive supranuclear palsy (PSP) (1, 5–7). In advanced PD and PSP, recurrent falls further impair functional independence and overall well-being (1). Consequently, predicting, preventing, and detecting falls at an early stage is an important therapeutic goal for patients with these disorders (5).

Despite their clinical importance, accurate identification and effective prevention strategies for falls in PD and PSP remain insufficiently established (1). Conventional assessment methods rely mainly on self-reporting or caregiver observation, which can result in underreporting and recall bias (8). Real-time, objective detection of falls remains a challenge in daily clinical practice. Existing fall detection approaches, including wearable and vision-based systems, have shown promising results, however, many rely on simulated falls or lack validation in real-world Parkinsonian populations. Recent advances in body landmark tracking and computer vision have improved detection accuracy, but their applicability to spontaneous falls in Parkinsonian syndromes remains unclear (9–11).

In recent years, the application of artificial intelligence (AI) technologies to clinical symptom analysis has gained increasing attention, offering objective and scalable quantification (12–14). Recent AI developments, including lightweight deep learning and spiking neural network approaches, have further expanded the applicability of computer vision methods in healthcare-related image analysis (15). In this study, we evaluated the feasibility of a privacy-preserving, non-wearable AI-based fall detection system using real-world continuous monitoring in an aged-care facility specializing in PD and atypical Parkinsonian syndromes. Unlike many previous studies based on simulated falls or wearable devices, our approach focused on spontaneous falls occurring in routine clinical environments. The system was designed to identify falls in real-time, enabling prompt response and care. Additionally, we performed a video-based analysis to describe falls in terms of frequency, timing, location, and circumstances, and to explore potential disease-related patterns.

## Methods

### Study design and protocol

This study was conducted between October 2022 and October 2023. After obtaining consent, cognitive functions were assessed using the MMSE, and ADL were assessed using the Barthel Index (BI). The participants resided in rooms equipped with video monitoring and were video recorded for 22–63 consecutive days. Of the total 6,424 h of raw recordings, 152 h were excluded *a priori* due to room entry/exit or system malfunction according to predefined criteria, leaving 6,272 h of valid recording time for analysis. No imputation was performed. Each room measured 5.2 m (depth) × 2.6 m (width) × 2.5 m (height). We used a video graphics array (VGA) and depth camera (GLORY, Hyogo, Japan) that employed near-infrared (NIR) technology, enabling fall detection regardless of the lighting conditions. Unlike conventional cameras, this device emits NIR light and captures its reflection using a dedicated sensor to produce greyscale images. The VGA camera recorded 640 × 480 pixels at 5 fps (frames per second). It was installed on the ceiling along the centreline of the room, approximately 1 m from the wall opposite the entrance. The distance data from the camera to the surrounding objects were incorporated into the analyses of posture and movement. The camera angle was automatically adjusted by a motor based on the image recognition of the human position. Recorded depth-camera images were processed locally using a proprietary, pre-trained human body posture-estimation model implemented through in-sensor edge computing (16). The model detected 18 key body points and generated skeletal representations together with depth information. The fall-detection pipeline comprised local extraction of skeletal key points, temporal assessment of skeletal movement and postural transitions, and generation of a fall alert when the proprietary decision criteria were met (Figure 1). The algorithm update primarily addressed failure modes observed during real-world monitoring, including poor subject extraction from infrared images and misalignment of skeletal key points. The cameras were operated continuously for 24 h/day. This study comprised two distinct but complementary components: (1) real-world evaluation and refinement of the AI-based fall detection system, and (2) video-based characterization of all video-confirmed fall events. For evaluation of the AI system, recordings from the first four participants were used for the initial real-world assessment. Based on the detection failures and technical issues identified during this phase, the algorithm was updated. The updated algorithm was then fixed and evaluated using recordings from the remaining participants, whose data had not been used for the algorithm update. Separately from the evaluation of AI detection performance, all video-confirmed falls from both phases were included in the analysis of fall characteristics, regardless of whether they had been detected by the AI system or reported by facility staff. In parallel, facility staff documented falls using conventional methods, including direct observation, responses to loud noises suggestive of a fall, and participant self-reports, and recorded the date and time of each confirmed event.

**Figure 1:**
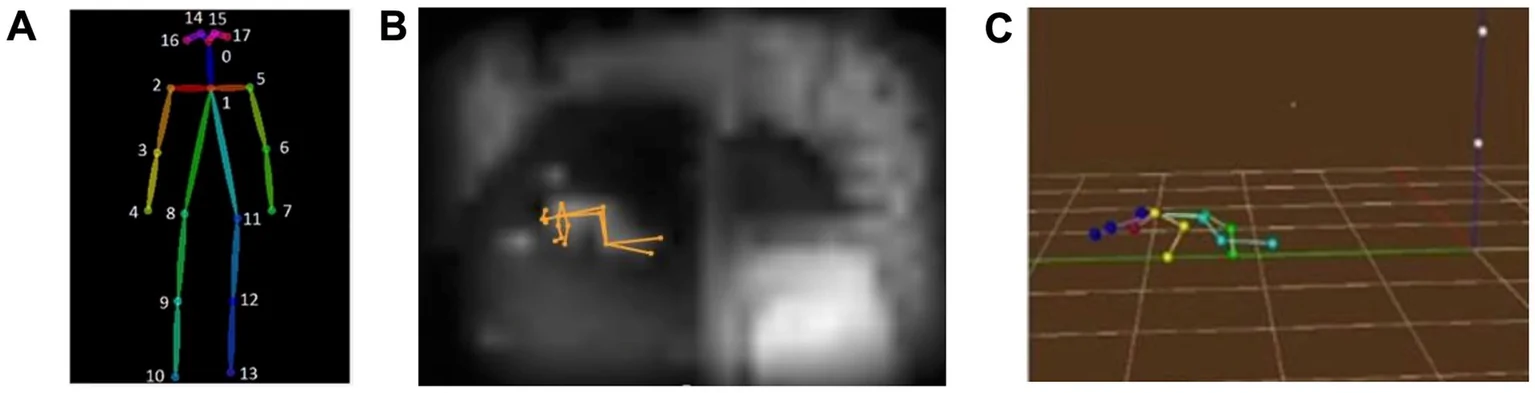
Real-time fall detection using skeletal tracking and AI algorithms. **(A)** Skeletal lines from 18 key body points detected via human pose estimation. **(B)** Real-time fall detection using edge AI analysing skeletal motion to classify posture transitions. **(C)** 3D skeletal data visualization corresponding to **(B)** for analysis.

### Study subjects

We included residents of an aged-care facility specialising in PD and atypical Parkinsonian syndromes (PD House, Sunwels Ltd., Japan). The inclusion criteria were: (1) age ≥ 20 years; (2) residence in a PD House (PD House Itabashi, Japan); (3) diagnosis of PD, multiple system atrophy (MSA), PSP, or corticobasal syndrome (CBS) according to the clinical diagnostic criteria (17–20); (4) history of at least one fall in the past year; and (5) provision of written informed consent. The exclusion criteria were severe dementia (Mini-Mental State Examination [MMSE] score < 10), a lack of capacity to understand the study and provide informed consent. The sample size was determined by participant availability during recruitment and was not based on statistical power calculations.

### Fall analysis

Two independent researchers (HK and KN), blinded to the AI detection results and facility reports, reviewed all videos to count the number of falls. Falls detected by the AI system and those recorded by the facility were counted, and sensitivity and positive predictive value were summarized descriptively for each method. For each video-recorded fall, the anonymised footage was reviewed independently by two researchers who documented findings in seven categories: time zone, position, action, direction, site of injury, primary category, and additional categories. Time was defined as morning (05:00–12:00), afternoon (12:00–17:00), evening (17:00–22:00), and night (22:00–05:00). The pre-fall positions were sitting, semi-squatting, or standing. An action describes an activity performed at that time. The directions were defined as forward, lateral, or backward. Based on previous classifications, falls were categorized as inadequate weight shift, trip or stumble, bump, loss of consciousness, slip, and/or loss of support (8, 21). We additionally documented four PD-related categories: dyskinesia, abnormal posture, freezing of gait (FOG)/festination, and attention/executive dysfunction (including visuospatial deficits) (22–24). This yielded 10 final categories (Table 1). Video-based categories represent rater-inferred, operational labels based on observable context; they do not constitute direct measurement of underlying physiology, cognition, or consciousness. Considering previous studies, an inadequate weight shift was defined as a seemingly self-induced shift in body weight, causing the center of gravity to move outside the base of support. This category differs from slip, trip, or stumble, and hit or bump in that no obvious external perturbation was identifiable on video review (8). Two researchers independently evaluated the categories of each fall and identified the primary and additional categories. For the analysis of primary categories, inter-rater agreement was calculated using the κ coefficient (25), and in cases of disagreement, a third researcher (GO) made the final decision. For the analysis of additional categories, all categories identified by either rater were included.

**Table 1:** Classification and definitions of fall categories.

Category	Description
Inadequate weight shift	A seemingly self-induced shifting of bodyweight causing the center of gravity to move outside the base of support. This is an internal cause of imbalance, distinct from slip, trip, stumble, or bump.
Trip or stumble	External perturbation caused by catching the foot on an object or uneven surface resulting in loss of balance.
Bump	Being hit or colliding with an object or person causing a loss of balance.
Loss of consciousness	Sudden collapse suggestive of possible loss of consciousness or fainting on video review.
Slip	External perturbation due to a slippery surface leading to loss of balance.
Loss of support	Loss of external support such as grabbing or holding an object or person for balance, which leads to falling.
Dyskinesia	Abnormal involuntary movements characteristic of PD that can contribute to falls.
Abnormal posture	Postural abnormalities often seen in PD patients that impair balance and increase fall risk.
FOG/festination	Gait disturbances specific to PD such as freezing of gait or festination that contribute to falls.
Attention and executive dysfunction	Observable behaviours compatible with possible attentional, executive, or visuospatial contributions to the fall, such as divided-attention activities, reaching or planning errors, or inappropriate wheelchair use.

Operational categories and definitions used for video-based fall analysis. Categories were assigned from observable video context and do not constitute direct measurements or diagnoses of the underlying physiological, neurological, or cognitive mechanisms.

### Statistical analysis

Statistical analyses were performed in R (version 4.3.2; R Foundation for Statistical Computing, Vienna, Austria). Sensitivity and positive predictive value (PPV) were calculated descriptively for both the AI-based system and conventional facility reporting. The corresponding 95% confidence intervals (CIs) were estimated using the Clopper–Pearson method based on the binomial distribution. No formal statistical comparison of detection rates was performed because multiple fall events originated from the same small number of participants and could not be regarded as independent observations. Specificity and negative predictive value were not calculated because continuous monitoring did not provide a predefined, clinically meaningful number of non-fall events that could serve as the true-negative denominator. To assess agreement between the two independent raters in identifying the primary category of falls, inter-rater reliability was calculated using Cohen’s kappa coefficient (κ). For the exploratory event-level analysis of categorical fall characteristics, chi-square (χ^2^) tests were used to assess departures from independence across seven variables: time zone, position, action, direction, site of injury, primary categories, and additional categories. For each variable, observed and expected counts were compared using a *χ*^2^ test, and multiplicity was controlled using the Benjamini–Hochberg false discovery rate (FDR) procedure. Standardised residuals (SRs) were calculated to identify the cells contributing most strongly to the overall *χ*^2^ statistic; |SR| ≥ 2 was considered to indicate a notable cell-level deviation. To compare fall characteristics between the PD and PSP groups, *χ*^2^ tests with FDR adjustment were applied to the same set of categorical variables. Standardised residuals were calculated to identify the categories contributing to the observed group differences. To explore whether individual participants tended to exhibit specific primary fall categories, the distribution of primary categories across participants was also analysed using a χ^2^ test. Because multiple falls were recorded from the same participants, the event-level observations were not completely independent. The limited number of participants contributing falls, particularly the inclusion of only two participants with PSP, did not permit reliable estimation using mixed-effects models or generalised estimating equations. Therefore, the analyses of fall characteristics were considered exploratory and hypothesis-generating, and the resulting *p*-values should be interpreted cautiously. A *p*-value < 0.05 was considered statistically significant. Continuous variables are summarised as median (interquartile range [IQR]). Categorical variables are shown as counts (%).

### Data and code availability

De-identified event-level fall data and an accompanying data dictionary may be made available from the corresponding author upon reasonable request, subject to institutional approval and an appropriate data-use agreement. The trained posture-estimation model, fall-detection algorithm, inference code, and model-evaluation scripts contain proprietary intellectual property of the technology provider and therefore cannot be publicly shared. The overall processing workflow and all technical information permitted by the provider are described in the Methods. The study protocol and statistical analysis plan may also be made available upon reasonable request. This study was conducted and reported in accordance with the STROBE guidelines for observational studies.

## Results

### Patient characteristics

This study included seven patients (five males, two females; four with PD, two with PSP, and one with CBS). The participant with CBS was excluded from the fall analyses because no falls occurred during the observation period. Thus, the fall analyses included six participants (five males and one female; four with PD and two with PSP). Among these participants, the median age was 75.0 years (68.5–82.3), the median MMSE score was 28.0 (20.0–28.0; one missing value), and the median BI was 64.0 (49.5–71.8) (Table 2).

**Table 2:** Background characteristics of participants included in the fall analyses.

Pt. No	Age	Sex	Diagnosis	MMSE	BI	Complication
1	83	M	PSP	NA	11	Alcoholic brainstem cerebellar atrophy
2	80	M	PD	28	72	OPLL, SAH (post-surgery)
3	63	M	PSP	20	74	DM
5	88	F	PD	20	47	Femoral neck fracture
6	70	M	PD	28	71	Right knee fracture
7	68	M	PD	28	57	
	75 (68.5–82.3)	M = 5, *F* = 1		28 (20–28)	64 (49.5–71.8)	

Data are presented as median (IQR). Participant 4, who had CBS, was excluded from the fall analyses because no falls occurred during the observation period. MMSE, Mini-Mental State Examination; BI, Barthel Index; M, male; F, female; PSP, progressive supranuclear palsy; PD, Parkinson’s disease; NA, not applicable; OPLL, Ossification of the Posterior Longitudinal Ligament; SAH, Subarachnoid haemorrhage; DM, diabetes mellitus.

### Falls detected by AI versus the conventional method

A total of 267 observation days (6,424 h) were obtained, comprising 122 days (3,008 h) in the initial real-world evaluation and refinement phase and 145 days (3,416 h) in the post-update evaluation phase. Across all recordings, 75 falls were documented (Supplementary Video 1). One fall that occurred outside the camera’s field of view was excluded, leaving 74 falls for analysis. Of these, 40 occurred in the initial real-world evaluation and refinement phase and 34 in the post-update evaluation phase.

The AI-based detection system identified 39 of 74 falls, yielding an event-level sensitivity of 52.7% (95% CI, 40.7–64.4), whereas conventional facility reporting identified 21 of 74 falls, yielding an event-level sensitivity of 28.4% (95% CI, 18.5–40.1). These estimates are presented descriptively because multiple falls originated from the same participants. During the post-update evaluation phase, the AI system detected 23 of 34 falls, corresponding to an event-level sensitivity of 67.6% (95% CI, 49.5–82.6), whereas conventional facility reporting identified 10 of 34 falls, corresponding to an event-level sensitivity of 29.4% (95% CI, 15.1–47.5). The PPV for the AI system was 45.3% (95% CI 34.6–56.5) overall and 41.8% (95% CI 28.7–55.9) in the post-update phase, whereas the PPV for conventional reporting remained 100% (95% CI 83.9–100 overall; 69.2–100 post-update).

### Descriptive characteristics of falls

Among the 74 fall events analysed, most occurred in the evening (36.5%) and afternoon (27.0%). Falls were most frequently observed in the standing position (51.4%) and were commonly associated with reaching (29.7%) or ambulation (20.3%). The majority of falls occurred backward (73.0%), with the gluteal region being the most frequent site of injury (77.0%). Detailed distributions are provided in Supplementary Table 1.

### Classification and analysis of primary and contributing categories of falls

Two independent researchers categorized primary and additional categories for each fall using the 10 predefined categories (Table 1) and the inter-rater agreement between the two independent raters was excellent, with a percent agreement of 97.3% (κ = 0.96, *p* < 0.001). The most frequently assigned primary operational categories were attention/executive dysfunction (43.2%) and inadequate weight shift (41.9%). Among the additional operational categories, inadequate weight shift (48.6%) and attention/executive dysfunction (27.0%) were most frequently assigned.

### Differences in fall categories and fall types based on patient attributes

In exploratory event-level analyses of the 74 fall events (PD, *n* = 54; PSP, *n* = 20), the overall distributions of time zone, primary category, and additional categories differed between the diagnostic groups (FDR-adjusted *p* = 0.033, *p* = 0.033, and *p* = 0.014, respectively; Table 3). Morning falls were over-represented in PSP (standardised residual for PD, −3.22), whereas evening falls were numerically more common in PD but did not reach the predefined residual threshold (standardised residual, +1.79). Attention/executive dysfunction was over-represented as a primary operational category in PD (standardised residual, +2.59), whereas inadequate weight shift was over-represented in PSP (standardised residual for PD, −2.98). Among additional categories, inadequate weight shift was over-represented in PD (standardised residual, +2.44), whereas loss of support was over-represented in PSP (standardised residual for PD, −2.67; Figure 2). Within the PD group, the distribution of primary fall categories varied across individuals (exploratory χ^2^ test, *p* < 0.05).

**Table 3:** Fall characteristics in PD and PSP patients.

Characteristic	Category	PD (*n* = 54)		PSP (*n* = 20)		𝜒^2^	*p*-value (FDR)	Standardised residuals
		Count	%	Count	%			
Time zone	Morning	6	40	9	60			−3.220
	Afternoon	15	75	5	25	11.005	0.033	0.239
	Evening	23	85.2	4	14.8			1.793
	Night	10	83.3	2	16.7			0.883
Position	Semi-squat	13	86.7	2	13.3	2.531	0.282	1.337
	Sitting	16	76.2	5	23.8			0.392
	Standing	25	65.8	13	34.2			−1.430
Action	Ambulation	10	66.7	5	33.3	12.497	0.072	−0.616
	Bending/Sitting down	8	88.9	1	11.1			1.147
	Dressing	4	100	0	0			1.251
	Reaching	19	86.4	3	13.6			1.687
	Repositioning in a wheelchair	2	100	0	0			0.873
	Sit-to-stand	7	58.3	5	41.7			−1.248
	Transferring	4	40	6	60			−2.525
Direction	Backward	41	75.9	13	24.1	3.226	0.220	0.940
	Forward	6	85.7	1	14.3			0.798
	Lateral	7	53.8	6	46.2			−1.710
Site of injury	Gluteal region	43	75.4	14	24.6	4.746	0.115	0.875
	Hemibody	4	44.4	5	55.6			−2.056
	Upper limb	7	87.5	1	12.5			0.980
Primary category	Abnormal posture	1	100	0	0	13.869	0.033	0.613
	Attention and executive dysfunction	29	87.9	4	12.1			2.590
	Dyskinesia	5	100	0	0			1.409
	Inadequate weight shift	17	54.8	14	45.2			−2.983
	Loss of support	0	0	1	100			−1.654
	FOG/festination	2	66.7	1	33.3			−0.251
Additional categories	Abnormal posture	10	100	0	0	18.318	0.014	1.731
	Attention and executive dysfunction	10	58.8	7	41.2			−2.291
	Inadequate weight shift	33	91.7	3	8.3			2.437
	Loss of support	6	50	6	50			−2.669
	FOG/festination	4	66.7	2	33.3			−0.774
	Trip or Stumble	5	100	0	0			1.185

Fall characteristics analysed across seven categories; time, position, action, direction, injury site, primary category, and additional categories in PD and PSP. Group differences were evaluated using exploratory event-level analyses. Because multiple falls originated from the same participants, the analyses did not account for within-participant correlation and the results should be interpreted cautiously. The values shown as percentages are row percentages within each fall characteristic or category; *n* denotes the number of fall events. PD, Parkinson’s disease; PSP, progressive supranuclear palsy; FDR, false discovery rate.

**Figure 2:**
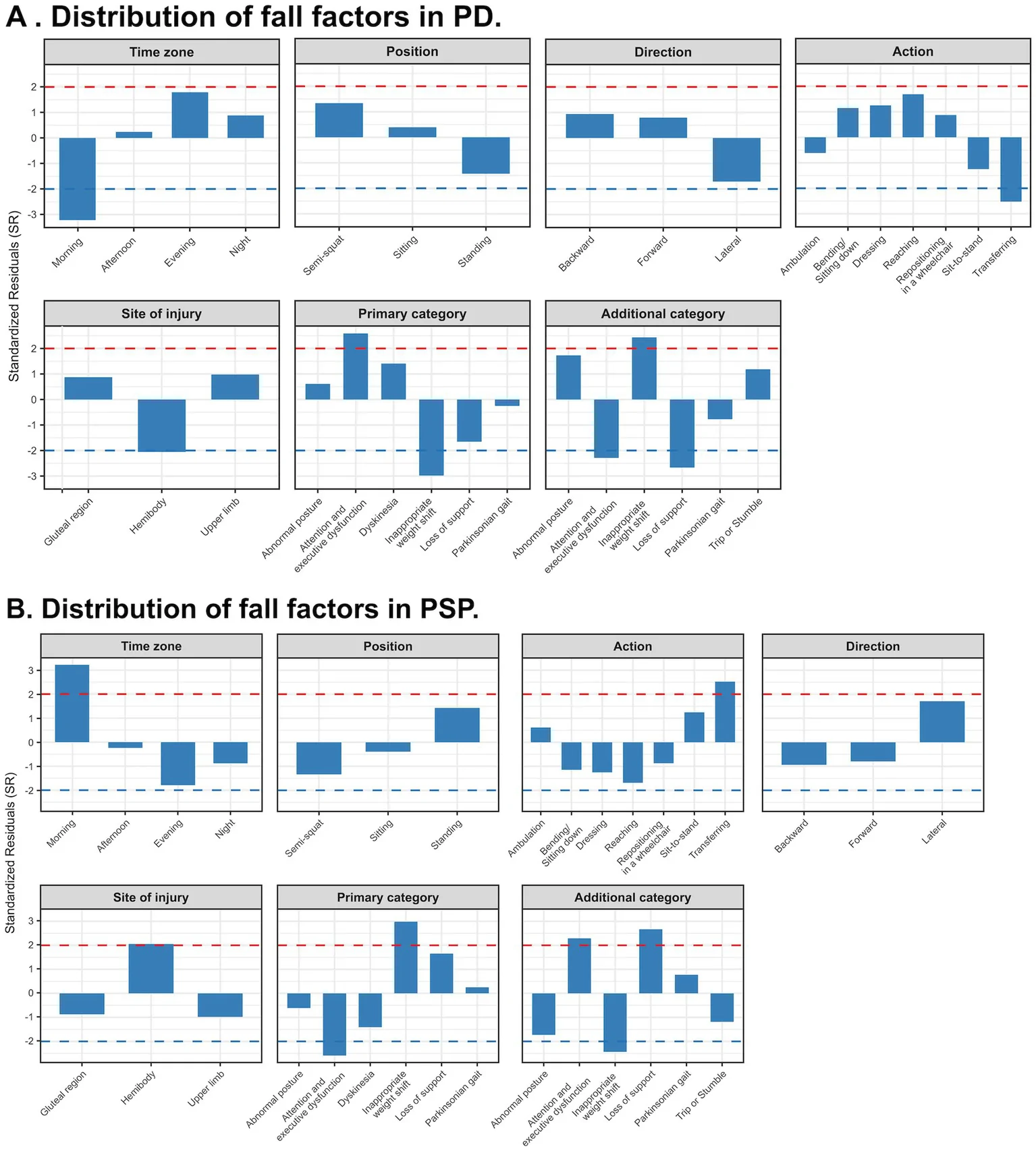
Distribution of fall categories in PD and PSP. Standardized residuals (SRs) for categorical fall characteristics in PD **(A)** and PSP **(B)**. SRs were computed for seven variables. The *x*-axis shows categories, and the *y*-axis SRs. Red and blue dashed lines at ±2 indicate reference thresholds for notable standardised residuals.

## Discussion

In this study, we evaluated the feasibility and practical applicability of a real-time fall detection system deployed in a nursing home, which uses AI to analyze depth camera footage while maintaining privacy protection. We evaluated the detection performance of the AI-based system against conventional facility reporting and separately characterized the circumstances and operational categories of all video-confirmed falls. AI-based fall detection showed numerically higher event-level sensitivity than conventional facility reporting and identified falls that had not been captured during routine daily care. Continuous non-wearable monitoring may therefore provide clinically important information that is difficult to capture using conventional self-report– or staff-dependent approaches. In addition to event detection, video analysis enabled characterization of spontaneous fall behaviors and contextual features that are difficult to assess using wearable sensors alone. Falls in PD were more often classified by the video raters as showing observable contexts compatible with attentional, executive, or visuospatial contributions during multitasking or turning behaviors, whereas falls in PSP were more often classified as involving inadequate weight shift or transfer-related instability. These video-based operational classifications do not establish the underlying neurological or cognitive mechanism of an individual fall. Moreover, because multiple events originated from only six participants, the observed disease-related patterns should be regarded as exploratory and hypothesis-generating.

Approximately 40–70% of patients with PD experience falls each year, and one-third of these individuals experience recurrent falls (26–28). This fall rate is twice that of the general elderly population, indicating that PD patients are at a significantly higher risk of falls (29). Falls not only present direct risks such as death, fractures, and hospitalization but also lead to secondary effects, such as reduced mobility and social isolation due to the fear of falling. These secondary effects often outweigh the direct consequences (30). For PD patients, particularly in the advanced to terminal stages, falls are one of the most critical symptoms that need to be managed. Falls in PD patients are known to be caused by various factors, including postural instability, FOG, inattention or cognitive decline, lower limb muscle weakness, orthostatic hypotension, and abnormal posture (31). However, there is limited data on the real-world frequency and nature of falls in PD patients.

Falls are typically self-reported, but self-reporting is prone to underreporting or inaccuracies due to memory errors, resulting in an actual incidence of falls that is higher than self-reported falls (32). This is particularly problematic in patients with PD, where the underreporting of falls has been documented. In our study, conventional facility reporting, including direct observation, responses to fall-related noises, and participant self-reports, captured only 28.4% of video-confirmed falls, indicating that more than two-thirds were not identified through routine reporting. Additionally, it is possible that some fall events were overlooked because, by the time staff arrived at the scene, the patient had already recovered their posture, or a time lag occurred before the fall was discovered. To address these issues of reliance on self-reporting and underreporting, digital devices that enable symptom monitoring independent of patient reports have gained attention, and in recent years, various such devices have been increasingly applied to PD and other neurodegenerative diseases (12, 33–35). Wearable sensors, smartphones, smart speakers, and vision-based systems are increasingly being used for fall detection in patients with PD. (13, 34–38) Recent vision-based approaches using body landmark tracking, posture classification, and threshold-based movement analysis have reported high accuracy in controlled datasets; however, many remain dependent on simulated falls or laboratory environments (9–11). Wearable devices, such as inertial measurement unit-based systems, sensor-integrated footwear, and full-body motion capture, have been most widely studied, with reported detection sensitivities ranging from 70% to 90% (36–39). Smartphone-based approaches, including waist-mounted devices and remote monitoring applications, have shown promise for postural stability assessment and fall risk estimation, but require strict placement compliance and have limited validation in actual PD or atypical Parkinsonian syndrome populations (40, 41). Accurately estimating the total number of falls remains inherently challenging. In one study, wearable devices detected approximately 70% of all falls. However, participants did not report these events, making it difficult to determine whether this was due to underreporting or false detection (42). Furthermore, conventional studies on fall detection in PD have been limited by the absence of standardization and insufficient real-world validation (36, 43). Many previous studies have relied on simulated falls in healthy individuals rather than spontaneous falls in patients, raising concerns about external validity. In addition, most device-based approaches require self-reporting to verify whether a fall has truly occurred, leaving considerable uncertainty in their validation accuracy. Unlike many previous studies using simulated falls or controlled datasets, the present study evaluated spontaneous falls occurring during continuous real-world monitoring of patients with advanced Parkinsonian syndromes. Previous wearable- and vision-based fall-detection studies have varied substantially in study populations, use of simulated versus real-world fall data, sensing modalities, validation designs, and reported performance metrics (Supplementary Table 2) (44–51). Direct numerical comparisons should nevertheless be interpreted cautiously because definitions of falls, true-negative observation windows, validation designs, and reported performance metrics differed across studies. In the present study, comprehensive video review likewise demonstrated the limitations of routine facility reporting and provided a more complete reference count of confirmed falls within the analyzable recordings. Although the algorithm was initially developed using simulated falls, it was subsequently refined to address real-world challenges, including poor subject extraction from infrared images and misalignment of skeletal key points. The updated algorithm showed numerically higher sensitivity during the post-update evaluation phase. However, because different participants were included before and after the update, the observed difference cannot be attributed solely to the algorithm modification. Sensitivity and PPV were selected as the principal performance metrics because they directly reflect the proportion of confirmed falls detected and the proportion of generated alerts corresponding to actual falls. Specificity and negative predictive value could not be meaningfully estimated because continuous monitoring did not provide a predefined true-negative event denominator. The PPV of the AI system was moderate, ranging from 41.8% to 45.3%, indicating that more than half of the alerts did not correspond to video-confirmed falls. Although prioritizing sensitivity may be appropriate for a safety-oriented screening system, false alarms may increase caregiver workload and reduce confidence in the system. Further refinement will therefore be required to reduce false-positive alerts while maintaining clinically useful sensitivity.

Robinovitch et al. (8) were the first to observe and analyze falls in elderly residents of long-term care facilities using video analysis. Their study revealed that 4% of falls occurred during walking, and 12% were due to interactions with others. A majority of the falls (80%) were forward falls, and 70% resulted in contact with the floor. However, this study focused on a general elderly population and included a variety of conditions such as dementia, post-stroke, and PD. Given that only 15 out of 271 participants had PD, the findings may not be directly applicable to patients with PD. Our system observed otherwise unrecognized falls, particularly at night or in private rooms. Moreover, the integration of video review enabled a more detailed descriptive characterization of falls, particularly with respect to observable contextual elements, such as environmental conditions and specific movements, that are not detectable by wearable devices (52). In our facility-based cohort, backward falls and falls occurring in living areas were observed relatively often in PD, and these events were frequently classified into categories related to cognitive, attentional, and visuospatial features rather than to FOG. This pattern may reflect setting-specific factors and may differ from distributions reported in other PD populations. Several events appeared potentially amenable to simple environmental modifications, such as default-elevated footrests, auto-locking wheelchairs, and caregiver interventions. Future studies should examine whether these observable video-based patterns are associated with formal neuropsychological measures of attention and visuospatial function. In PSP, falls predominantly occurred during transfers and were more frequently classified as involving inadequate weight shift, consistent with postural instability and potentially compounded by motor and functional decline. Across both diseases, inadequate weight shift was frequently observed, often co-occurring with observable behaviours assigned to the attention/executive dysfunction category, such as inappropriate wheelchair use or reaching errors during multitasking, rather than occurring alone. Patients with FOG or abnormal posture were frequently represented among fall events. Unlike community-dwelling older adults, falls from stumbling, tripping, or bumping were rare in this study, suggesting that prevention strategies may need to be tailored to Parkinsonian syndromes (8).

This study has several important limitations. First, the number of participants was small, and only six participants contributed fall events. Although 74 falls were available for event-level analysis, multiple events originated from the same individuals and therefore could not be regarded as fully independent observations. Patient-specific characteristics or recurrent fall patterns may consequently have influenced the apparent disease-group differences. Because the number of participant-level clusters was too small for reliable mixed-effects modelling or generalised estimating equations, these comparisons should be regarded as exploratory and hypothesis-generating. In addition, disease subtypes within PD and PSP were not analysed, and pathological confirmation was not available; therefore, diagnostic heterogeneity cannot be excluded. Second, the study population consisted exclusively of residents of a PD-specialised long-term care facility. These individuals are likely to have more advanced disease, greater functional impairment, and a higher burden of comorbidities than community-dwelling patients, introducing potential selection bias and limiting generalisability to other clinical settings. Accordingly, the observed fall patterns and detection performance may not be directly generalizable to community-dwelling patients, independently living patients, or individuals with earlier-stage disease, in whom fall frequency, daily activities, environmental conditions, and caregiver availability may differ. Third, several clinically relevant factors known to influence fall risk were not systematically assessed, including medication use, such as benzodiazepines and levodopa equivalent daily dose, autonomic dysfunction including orthostatic hypotension, and standard measures of disease severity including Hoehn and Yahr stage or UPDRS scores. In addition, no concurrent physiological monitoring (such as blood pressure or electroencephalography) was performed, limiting the ability to determine whether individual falls were associated with syncope, transient loss of consciousness, or other physiological events. Fourth, the classification of falls was based on visual review of video recordings by expert raters. Although inter-rater agreement was high, the assignment of fall categories, particularly those related to cognitive or attentional features, remains inherently interpretative and subjective, as such features cannot be directly measured from video data alone. Accordingly, the classification results should be regarded as descriptive and exploratory rather than as evidence of causal or pathophysiological mechanisms. We did not use public benchmark datasets because many are based on simulated falls in healthy volunteers. Our aim was to evaluate spontaneous real-world falls in patients with Parkinsonian syndromes. Finally, different participants were included in the initial and post-update phases, precluding separation of algorithm-related effects from participant-related differences.

In the present study, AI-based video analysis was used to evaluate the feasibility of real-time fall detection and to describe fall-related characteristics. Falls in PD remain difficult to predict or prevent because of their multifactorial complexity (1, 29, 52). Our findings suggest that falls in both PD and PSP often involve multiple, patient-specific contributing categories. This observation supports the hypothesis that prevention strategies may need to consider both individual factors and diagnostic context, which should be tested in larger, multi-setting studies. Further validation using larger multi-center cohorts, multimodal wearable integration, and prospective real-world monitoring will be necessary to determine the generalizability and clinical utility of these findings (53–55). AI-based motion analysis integrated with patient background information may provide a framework for more individualized fall risk assessment in real-world clinical settings.

## Data Availability

De-identified event-level data supporting the conclusions of this article may be made available by the corresponding author upon reasonable request, subject to institutional approval and an appropriate data-use agreement. Proprietary models, algorithms, and associated code cannot be publicly shared.

## Supplementary material

SUPPLEMENTARY VIDEO 1 Video of real-time fall detection using AI-based skeletal posture analysis. Video processed with a proprietary pose estimation model via edge AI. Eighteen body key points formed skeletal lines for real-time fall detection. The clip shows a wheelchair user reaching forward then falling at 10 s. Click to play video.
