# Supplementary material for: Real-world feasibility of privacy-preserving, non-wearable AI for real-time fall detection with disease-specific video classification in parkinsonian syndromes: a proof-of-concept clinical study
Source: Front Neurol. 2026 Sep 18;17:1856113. doi: 10.3389/fneur.2026.1856113 (PMC13631938; doi:10.3389/fneur.2026.1856113)
Supplement: Supplementary file 1 [file Table_1.DOCX]

**Supplementary Table S1. Distribution of fall characteristics in the overall cohort**

| Pt No | Fall No | Time zone | Position | Action | Direction | Site of injury | Primary factor | Co-factors | | | Description |
| --- | --- | --- | --- | --- | --- | --- | --- | --- | --- | --- | --- |
| 1 | 1 | Morning | Sitting | Sit-to-stand | Backward | Gluteal region | Inappropriate weight shift | Loss of support |  |  | Backward fall when rising from bed |
|  | 2 | Morning | Sitting | Sit-to-stand | Backward | Gluteal region | Inappropriate weight shift | Loss of support |  |  | Backward fall when rising from bed |
|  | 3 | Afternoon | Sitting | Reaching | Lateral | Hemibody | Inappropriate weight shift | Attention and executive dysfunction |  |  | Slid off the wheelchair when trying to get something out |
|  | 4 | Afternoon | Standing | Ambulation | Backward | Hemibody | Inappropriate weight shift | Parkinsonian gait |  |  | Tripping and falling |
|  | 5 | Morning | Sitting | Sit-to-stand | Backward | Gluteal region | Inappropriate weight shift | Loss of support |  |  | Backward fall when rising from bed |
|  | 6 | Morning | Standing | Transferring | Lateral | Hemibody | Inappropriate weight shift | Loss of support |  |  | Fall onto bed during transfer from wheelchair |
|  | 7 | Morning | Standing | Ambulation | Backward | Gluteal region | Inappropriate weight shift | Parkinsonian gait | Attention and executive dysfunction |  | Fell without support while walking |
|  | 8 | Morning | Standing | Sit-to-stand | Backward | Gluteal region | Attention and executive dysfunction | Inappropriate weight shift |  |  | Fall during transfer from wheelchair |
|  | 9 | Afternoon | Standing | Transferring | Lateral | Hemibody | Inappropriate weight shift | Loss of support | Attention and executive dysfunction |  | Losing balance and falling when transferring |
|  | 10 | Evening | Standing | Transferring | Lateral | Hemibody | Loss of support | Attention and executive dysfunction |  |  | Losing balance and falling when transferring |
|  | 11 | Night | Standing | Ambulation | Forward | Upper limb | Parkinsonian gait | Inappropriate weight shift |  |  | Forward fall onto bed due to festination while walking to sit |
|  | 12 | Morning | Standing | Reaching | Backward | Gluteal region | Inappropriate weight shift | Attention and executive dysfunction |  |  | Losing balance and falling when transferring |
|  | 13 | Afternoon | Standing | Sit-to-stand | Lateral | Gluteal region | Inappropriate weight shift | Loss of support |  |  | Lateral fall when rising from bed |
|  | 14 | Morning | Standing | Transferring | Backward | Gluteal region | Attention and executive dysfunction |  |  |  | Losing balance and falling when transferring |
|  | 15 | Evening | Standing | Ambulation | Backward | Gluteal region | Inappropriate weight shift |  |  |  | Fall during ambulation around bed |
|  | 16 | Morning | Sitting | Transferring | Backward | Gluteal region | Attention and executive dysfunction |  |  |  | Fall from failed transfer when sitting onto wheelchair |
|  | 17 | Evening | Standing | Transferring | Lateral | Gluteal region | Inappropriate weight shift |  |  |  | Losing balance and falling when transferring |
|  | 18 | Night | Standing | Ambulation | Backward | Gluteal region | Inappropriate weight shift | Attention and executive dysfunction |  |  | Fell without support while walking |
| 2 | 1 | Evening | Standing | Bending/Sitting down | Backward | Gluteal region | Attention and executive dysfunction |  |  |  | Fall due to missed seating in wheelchair, living room |
|  | 2 | Evening | Standing | Reaching | Backward | Gluteal region | Inappropriate weight shift |  |  |  | Fall from loss of balance while reaching into dresser |
|  | 3 | Afternoon | Standing | Ambulation | Backward | Gluteal region | Inappropriate weight shift |  |  |  | Backward fall from loss of balance while walking near bed |
|  | 4 | Afternoon | Standing | Ambulation | Backward | Gluteal region | Inappropriate weight shift |  |  |  | Backward fall from loss of balance while walking near desk |
|  | 5 | Evening | Sitting | Reaching | Forward | Upper limb | Attention and executive dysfunction |  |  |  | Forward slide from wheelchair when reaching to floor (wheelchair unlocked) |
|  | 6 | Night | Standing | Sit-to-stand | Backward | Gluteal region | Inappropriate weight shift |  |  |  | Backward fall after standing up quickly from crouching to pick up object |
|  | 7 | Morning | Sitting | Reaching | Backward | Gluteal region | Attention and executive dysfunction | Inappropriate weight shift |  |  | Slid off the wheelchair when trying to get something out. |
|  | 8 | Afternoon | Sitting | Reaching | Backward | Gluteal region | Attention and executive dysfunction | Inappropriate weight shift |  |  | Slid off the wheelchair when trying to get something out. |
|  | 9 | Afternoon | Sitting | Reaching | Backward | Gluteal region | Attention and executive dysfunction |  |  |  | Fall when sliding while backing wheelchair |
|  | 10 | Afternoon | Sitting | Reaching | Backward | Gluteal region | Attention and executive dysfunction | Inappropriate weight shift |  |  | Slid off the wheelchair when trying to get something out. |
|  | 11 | Afternoon | Standing | Ambulation | Backward | Gluteal region | Inappropriate weight shift | Loss of support |  |  | Held by a support, but lost balance and fell over. |
|  | 12 | Evening | Standing | Reaching | Backward | Gluteal region | Inappropriate weight shift | Loss of support | Attention and executive dysfunction |  | Backward fall after letting go of handrail while reaching |
|  | 13 | Evening | Standing | Ambulation | Backward | Gluteal region | Inappropriate weight shift | Loss of support |  |  | Held by a support, but lost balance and fell over. |
|  | 14 | Evening | Standing | Ambulation | Backward | Gluteal region | Attention and executive dysfunction | Inappropriate weight shift | Trip or Stumble |  | Held by a support, but lost balance and fell over. |
|  | 15 | Afternoon | Standing | Ambulation | Backward | Gluteal region | Inappropriate weight shift |  |  |  | Fall during ambulation around bed |
|  | 16 | Morning | Semi-squat | Bending/Sitting down | Backward | Gluteal region | Attention and executive dysfunction | Inappropriate weight shift |  |  | Squatting and falling when picking things up |
|  | 17 | Night | Standing | Ambulation | Backward | Gluteal region | Attention and executive dysfunction | Inappropriate weight shift |  |  | Fell without support while walking |
|  | 18 | Afternoon | Standing | Dressing | Backward | Gluteal region | Attention and executive dysfunction | Inappropriate weight shift | Loss of support |  | Held by a support, but lost balance and fell over. |
|  | 19 | Evening | Sitting | Repositioning in a wheelchair | Backward | Gluteal region | Attention and executive dysfunction |  |  |  | Wheelchair moves during re-sitting and falls over |
|  | 20 | Evening | Standing | Transferring | Backward | Gluteal region | Inappropriate weight shift | Attention and executive dysfunction | Parkinsonian gait |  | Losing balance and falling when transferring |
| 3 | 1 | Evening | Semi-squat | Bending/Sitting down | Backward | Gluteal region | Attention and executive dysfunction | Inappropriate weight shift |  |  | Squatting and falling when picking things up |
|  | 2 | Afternoon | Semi-squat | Reaching | Backward | Gluteal region | Inappropriate weight shift | Attention and executive dysfunction |  |  | Backward fall while reaching from semi-crouch |
| 5 | 1 | Afternoon | Standing | Transferring | Lateral | Hemibody | Attention and executive dysfunction | Inappropriate weight shift | Trip or Stumble | Parkinsonian gait | Tripping and falling |
|  | 2 | Night | Sitting | Bending/Sitting down | Backward | Gluteal region | Inappropriate weight shift |  |  |  | Wheelchair moves during re-sitting and falls over |
|  | 3 | Night | Semi-squat | Reaching | Backward | Gluteal region | Attention and executive dysfunction | Inappropriate weight shift |  |  | Squatting and falling when picking things up |
|  | 4 | Evening | Standing | Transferring | Backward | Gluteal region | Attention and executive dysfunction | Inappropriate weight shift | Trip or Stumble |  | Tripping and falling |
|  | 5 | Evening | Sitting | Repositioning in a wheelchair | Backward | Gluteal region | Attention and executive dysfunction | Inappropriate weight shift |  |  | Wheelchair moves during re-sitting and falls over |
|  | 6 | Morning | Semi-squat | Bending/Sitting down | Lateral | Gluteal region | Attention and executive dysfunction | Inappropriate weight shift |  |  | Squatting and falling when picking things up |
|  | 7 | Evening | Sitting | Reaching | Backward | Gluteal region | Attention and executive dysfunction | Inappropriate weight shift |  |  | Slid off the wheelchair when trying to get something out. |
|  | 8 | Evening | Sitting | Reaching | Backward | Gluteal region | Attention and executive dysfunction | Inappropriate weight shift |  |  | Slid off the wheelchair when trying to get something out. |
|  | 9 | Evening | Standing | Reaching | Backward | Gluteal region | Dyskinesia | Inappropriate weight shift | Attention and executive dysfunction |  | Backward fall while reaching in semi-crouched position |
|  | 10 | Afternoon | Semi-squat | Bending/Sitting down | Lateral | Gluteal region | Attention and executive dysfunction | Inappropriate weight shift | Abnormal posture |  | Squatting and falling when picking things up |
|  | 11 | Evening | Semi-squat | Reaching | Backward | Gluteal region | Dyskinesia | Inappropriate weight shift | Attention and executive dysfunction |  | Backward fall while reaching in semi-crouched position |
|  | 12 | Evening | Semi-squat | Reaching | Backward | Gluteal region | Dyskinesia | Inappropriate weight shift | Attention and executive dysfunction |  | Backward fall while reaching in semi-crouched position |
|  | 13 | Afternoon | Semi-squat | Reaching | Backward | Gluteal region | Dyskinesia | Inappropriate weight shift | Attention and executive dysfunction |  | Backward fall while reaching in semi-crouched position |
|  | 14 | Evening | Sitting | Sit-to-stand | Lateral | Upper limb | Attention and executive dysfunction | Inappropriate weight shift | Trip or Stumble |  | Fall from tripping on wheelchair footrest |
|  | 15 | Evening | Semi-squat | Reaching | Backward | Gluteal region | Inappropriate weight shift | Attention and executive dysfunction |  |  | Backward fall while reaching in semi-crouched position |
|  | 16 | Evening | Semi-squat | Reaching | Backward | Gluteal region | Inappropriate weight shift | Attention and executive dysfunction |  |  | Backward fall while reaching in semi-crouched position |
|  | 17 | Night | Semi-squat | Reaching | Backward | Gluteal region | Inappropriate weight shift | Attention and executive dysfunction |  |  | Backward fall while reaching in semi-crouched position |
|  | 18 | Morning | Semi-squat | Dressing | Backward | Gluteal region | Attention and executive dysfunction | Inappropriate weight shift |  |  | Fall when attempting to change clothes |
|  | 19 | Evening | Sitting | Sit-to-stand | Lateral | Hemibody | Attention and executive dysfunction | Inappropriate weight shift |  |  | Losing balance and falling while changing clothes |
|  | 20 | Evening | Standing | Ambulation | Forward | Upper limb | Dyskinesia | Inappropriate weight shift | Attention and executive dysfunction | Abnormal posture | Fell due to FOG/festination |
|  | 21 | Night | Semi-squat | Bending/Sitting down | Backward | Gluteal region | Attention and executive dysfunction | Inappropriate weight shift | Abnormal posture |  | Squatting and falling when picking things up |
|  | 22 | Evening | Sitting | Sit-to-stand | Lateral | Hemibody | Attention and executive dysfunction | Inappropriate weight shift |  |  | Losing balance and falling while changing clothes |
|  | 23 | Afternoon | Sitting | Sit-to-stand | Forward | Upper limb | Attention and executive dysfunction | Inappropriate weight shift | Parkinsonian gait | Abnormal posture | Fell due to excessive momentum when standing up |
|  | 24 | Night | Standing | Reaching | Backward | Gluteal region | Attention and executive dysfunction | Inappropriate weight shift | Trip or Stumble |  | Squatting and falling when picking things up |
| 6 | 1 | Night | Semi-squat | Bending/Sitting down | Backward | Gluteal region | Inappropriate weight shift | Abnormal posture |  |  | Buttock fall while repositioning in wheelchair |
|  | 2 | Night | Standing | Ambulation | Forward | Upper limb | Parkinsonian gait | Inappropriate weight shift | Abnormal posture |  | Fell due to FOG/festination |
|  | 3 | Night | Standing | Sit-to-stand | Forward | Upper limb | Inappropriate weight shift | Abnormal posture |  |  | Fell due to excessive momentum when standing up |
|  | 4 | Morning | Sitting | Transferring | Forward | Upper limb | Abnormal posture | Inappropriate weight shift |  |  | Tried to get up from the bed and slipped off |
| 7 | 1 | Evening | Standing | Reaching | Lateral | Gluteal region | Inappropriate weight shift | Parkinsonian gait |  |  | Held by a support, but lost balance and fell over. |
|  | 2 | Evening | Standing | Ambulation | Backward | Gluteal region | Parkinsonian gait | Inappropriate weight shift | Abnormal posture |  | Held by a support, but lost balance and fell over. |
|  | 3 | Afternoon | Standing | Dressing | Backward | Gluteal region | Attention and executive dysfunction | Inappropriate weight shift |  |  | Losing balance and falling while changing clothes |
|  | 4 | Afternoon | Standing | Bending/Sitting down | Backward | Hemibody | Attention and executive dysfunction | Inappropriate weight shift | Loss of support | Abnormal posture | Losing balance and falling when transferring |
|  | 5 | Afternoon | Sitting | Sit-to-stand | Backward | Gluteal region | Attention and executive dysfunction | Inappropriate weight shift | Abnormal posture |  | Squatting and falling when picking things up |
|  | 6 | Morning | Standing | Dressing | Backward | Gluteal region | Inappropriate weight shift | Loss of support |  |  | Losing balance and falling while changing clothes |

Individual falls were classified according to their respective criteria. Fall times were categorized as morning (05:00–12:00), afternoon (12:00–17:00), evening (17:00–22:00), and night (22:00–05:00). Position prior to the fall was categorized as sitting, semi-squat, or standing. Action at the time of the fall were categorized as reaching, walking, transferring, bending/sitting down, dressing, sit-to-stand, or repositioning in a wheelchair. The direction of the fall was categorized as forward, backward, or sideward. The causes of falls were classified into 10 categories: inappropriate weight shift, trip or stumble, bumping, loss of consciousness, slipping, loss of support, dyskinesia, postural abnormalities due to Parkinson’s disease (PD), gait disturbance specific to PD, and impaired attention executive function and spatial cognition. Factors contributing to falls were evaluated separately by two independent researchers, and a list of factors and sub-factors was compiled. Pt, patient; No, number.
