# Supplementary material for: Real-world feasibility of privacy-preserving, non-wearable AI for real-time fall detection with disease-specific video classification in parkinsonian syndromes: a proof-of-concept clinical study
Source: Front Neurol. 2026 Sep 18;17:1856113. doi: 10.3389/fneur.2026.1856113 (PMC13631938; doi:10.3389/fneur.2026.1856113)
Supplement: Supplementary file 2 [file Table_2.docx]

**Supplementary Table 2. Comparison of representative wearable- and vision-based fall-detection studies**

| Study | Population / setting | Fall data | Sensor / input | Detection approach | Validation design | Reported performance | Key limitation |
| --- | --- | --- | --- | --- | --- | --- | --- |
| Boutellaa et al., 2019 | CogentLabs: 42 volunteers; DLR: 19 volunteers; controlled or semi-naturalistic settings | Scripted/simulated falls and ADLs; CogentLabs: 448 falls/1,520 ADLs; DLR: 56 falls/961 ADLs | Wearable accelerometers and gyroscopes at chest/thigh; accelerometer, gyroscope, and magnetometer at waist | Covariance-matrix feature extraction and sensor fusion with nearest-neighbour classification using Euclidean or Riemannian metrics | Within-dataset evaluation on two public datasets | Best accuracy: 92.51% (CogentLabs) and 98.31% (DLR); F1: 0.9598 and 0.9908 | No clinical patient cohort; predominantly scripted data; performance depends on dataset and sensor placement |
| Burwinkel et al., 2020 | 10 healthy adults aged 20–32 years; laboratory setting | 240 simulated falls, 240 near-falls, and 240 ADL trials | Binaural hearing-instrument IMUs; commercial pendant comparator | Two embedded firmware algorithms using acceleration, estimated falling distance, trunk orientation, and impact features | Repeated-measures paired comparison across three devices | Best hearing-instrument system: sensitivity 92.1%, specificity 98.8%, overall accuracy 96.5% | Young healthy participants and staged falls; no free-living or older-adult validation |
| Palmerini et al., 2020 | 40 individuals at moderate-to-high fall risk (mean age 69.2 years); community and geriatric rehabilitation settings | 143 spontaneous real-world falls; 180 h of real-world ADLs from 15 participants | Lower-back triaxial accelerometer | Multiphase fall-model features with SVM, logistic regression, kNN, random forest, and Naive Bayes | Stratified subject-based 5-fold cross-validation; nested tuning for SVM | Best SVM: sensitivity 81.1%, specificity 99.5%, PPV 53.7%, F1 64.6%, 0.56 false alarms/h | Fall identification relied on reports and signal review rather than continuous video ground truth; clinically relevant false-alarm burden |
| Saleh et al., 2021 | 16 long-term-care residents aged ≥80 years; 400 days of daily-life monitoring | Real-world ADLs and 2 spontaneous falls, supplemented with simulated falls from FallAllD | Neck- or wrist-worn accelerometer plus barometric pressure sensor | Rule-based preprocessing followed by rotation-invariant features and gradient-boosting classification | Subject-independent train/test split; test set included 303 days of real-world ADLs and 2 real falls | Sensitivity 100%; specificity 98.52% (neck) and 96.73% (wrist); ~1 false positive/25 days (neck) and /3 days (wrist) | Only two spontaneous falls; sensitivity was largely supported by simulated-fall data |
| Alizadeh et al., 2021 | Training: 23 healthy young adults in SisFall; external validation: 15 accidental falls from 15 older adults in FARSEEING | Intentional laboratory falls for training; spontaneous real-world falls for external testing | Waist/lower-back accelerometer | Linear SVM, kNN, and random forest using time- and frequency-domain features | Cross-dataset validation without retraining on FARSEEING | SVM on real-world test data: accuracy 96%, sensitivity 98%, specificity 94% (non-overlapping windows) | Small external real-world test set; manually segmented fall windows and limited evaluation of continuous false-alarm burden |
| Kothari & Chakurkar, 2025 | Publicly available pre-recorded video data; no newly recruited human participants | Custom video dataset containing staged fall events | RGB video | YOLOv8 person detection plus MediaPipe pose landmarks, bounding-box aspect ratio, temporal posture logic, and automated alerts | Frame-level comparison with ground truth on a pre-recorded video | Accuracy 96.06%, precision 75%, recall 100%, F1 75%; 6 ms/frame | Dataset size and population characteristics were not clearly reported; controlled/staged data and frame-level evaluation |
| Zobi et al., 2025 | Public Le2i and NTU RGB+D benchmark datasets; no new patient cohort | Staged falls and ADLs, with synthetic rotations and occlusions | Single-camera RGB video converted to 3D skeletal joint sequences using MMPose; Kinect data for cross-domain testing | Viewpoint-invariant graph convolutional network with synthetic viewpoint augmentation and inference-time aggregation | Non-overlapping held-out test subsets; cross-domain evaluation across Le2i and NTU RGB+D | Le2i: accuracy 99.81%, sensitivity 99.62%, specificity 100%, F1 99.81% | Controlled benchmark data with staged falls; very high performance may not translate to spontaneous clinical falls |
| Taheri et al., 2026 | UMAFall public benchmark; 13 participants after data cleaning (ages 18–55 years) | Simulated falls and scripted ADLs; 202 fall and 388 ADL trials after cleaning | Wrist IMU for student model; waist, wrist, ankle, and chest IMUs for teacher model | Knowledge-distilled lightweight CNN trained from a multi-location Transformer teacher | Two-subject held-out test plus supplementary leave-one-subject-out analysis | KD-CNN: accuracy 95.1%, precision 93.3%, recall 93.3%, F1 93.3%; LOSO AUC 0.96 ± 0.03 | Not evaluated in older adults or free-living conditions; performance based on a simulated benchmark |
| Present study | Six fall-contributing residents of a PD-specialised long-term-care facility (4 PD, 2 PSP; median age 75 years) | 74 spontaneous, video-confirmed real-world falls during 6,272 h of continuous monitoring | Ceiling-mounted near-infrared depth camera with in-sensor edge AI and 18-point skeletal representation | Proprietary pose estimation with temporal assessment of skeletal movement and postural transitions; blinded video adjudication | Initial real-world evaluation/refinement followed by fixed post-update evaluation in different participants | Sensitivity 52.7% overall and 67.6% post-update; PPV 45.3% overall and 41.8% post-update | Small cohort with repeated falls; moderate PPV; proprietary algorithm; limited generalisability beyond advanced Parkinsonian syndromes |

Studies were selected to illustrate differences in sensing modality, simulated versus real-world data, validation design, and clinically relevant performance metrics. Direct numerical comparisons should be interpreted cautiously because fall definitions, observation units, datasets, validation procedures, and metric definitions differed substantially across studies. ADL, activity of daily living; CNN, convolutional neural network; F1, F1 score; IMU, inertial measurement unit; kNN, k-nearest neighbours; PD, Parkinson’s disease; PPV, positive predictive value; PSP, progressive supranuclear palsy; SVM, support vector machine.
